# Prevalence of Risky Behaviors and Related Factors among Students of Dezful 

**Published:** 2017-07

**Authors:** Malihe Sohrabivafa, Mohammad Ali Tosang, Seyedeh Zeynab Molaei Zadeh, Elham Goodarzi, Zahra Sadat Asadi, Alireza Alikhani, Salman Khazaei, Seyedeh Leila Dehghani, Reza Beiranvand, Zaher Khazaei

**Affiliations:** 1 Department of Health and Community Medicine, Faculty of Medicine, Dezful University of Medical Sciences, Dezful, Iran.; 2 Social Development and Health Promotion Research Center, Gonabad University of Medical Sciences, Gonabad, Iran.; 3 Health Education & Promotion, Department of Community Medicine, School of Medicine, AJA University of Medical Sciences, Tehran, Iran.; 4 4 .Deputy of Medical Education Development Center, Kermanshah University of Medical Sciences, Kermanshah, Iran.; 5 Department of Epidemiology, School of Public Health, Hamadan University of Medical Sciences, Hamadan, Iran.; 6 Department of Public Health, Behbahan Faculty of Medical Sciences, Behbahan, Iran.; 7 Instructor of Epidemiology, Department of Epidemiology, Faculty of Medical Sciences, Shushtar, Iran.; 8 Social Determinants of Health Research Center, Kurdistan University of Medical Sciences, Sanandaj, Iran.

**Keywords:** *Dezful*, *Iran*, *Risky Behavior*, *Drug Abuse*, *Risky Sexual Behavior*, *Students*

## Abstract

**Objective:** There is a likelihood of risky behaviors such as drug abuse, risky sexual behavior, and adaptability issues in young ages. The present study aimed at investigating the prevalence of risky behaviors among students of Dezful University of Medical Sciences in 2014.

**Method**: This was a descriptive-analytical cross sectional study, with a random sampling approach. Scale of measuring risky behaviors was used to measure the risky behaviors (high speed driving, maim, drug use, and sexual behaviors) and related factors. The mean, standard deviation, Chi-square tests, t tests, and ANOVA were used for data analysis.

**Results:** The study was conducted on 150 (50%) female and 150 (50%) male students. Most of the participants aged 20 to 24 years. A statistically significant difference was obtained between the average scores of risky behaviors among female and male students (p˂0.05). The results of the present study revealed that the prevalence of risky behaviors, high speed driving, and drug consumption was different among the students of various study fields (p˂0.05).

**Conclusion:** The prevalence of risky behaviors among students of Dezful University was relatively low, and the prevalence of these behaviors in female students was far less than in male students. Risky behaviors were associated with background variables, except for mother's occupation.

Actually, university entrance is one of the most important stages of change and transition periods in life of young adults, 

which exposes them to an entirely new environment. These changes lead to increase in the stress level, compromise, and behavioral problems in students including risky behaviors (1). Risky behaviors are behavioral and social disorders that threaten social order. Despite the actions taken in the past 3 decades, these behaviors have grown exponentially worldwide (2). The prevalence of risky behaviors is one of the most serious factors threatening the health and psychological welfare of adolescents (3). 

Risky behaviors increases the possibility of destructive physical, psychological, and social consequences for the individual including the various behaviors such as poor diet, lack of physical activity, risky sexual behaviors, consumption of alcohol, tobacco, and drug, high speed driving, assaults, failing to wear a seat belt, and committing suicide or thinking about it (2). 

Risky behaviors are important in the life consequences, health, and psychological and social growth of the young (4). These behaviors increase the risk of premature death, disability, and increased incidence of chronic diseases (5). More than 50% of deaths from injuries occur in younger age groups (6).

Similar to our country, risky behaviors have had an upward trend in developing countries in the past 2 decades (7, 8). It is predicted that the rate of deaths caused by tobacco use in the world reach to 10 million per year by 2030, and if we take into account the physical, psychological, and social consequences of other risky behaviors such as drug abuse, violence, and risky sexual contacts, this number will be multiplied (9). Similar studies conducted in Turkey and Syria has also shown an increase in the rate of smoking in adolescents and those in younger ages (10). According to the results of one study, among the students of Tehran University, the prevalence of consuming hookah was 34%, cigarette 24%, alcohol 17%, opium 2.3%, cannabis 2.2%, and ecstasy pill 0.7%, respectively (11). In another study conducted on the prevalence of consumption of addictive drugs among students of various universities in Rasht, the prevalence of consuming cigarette was 24.3%, alcohol 10.5%, opium 4.87%, cannabis 2.2%, and ecstasy pill 7.25% (12). Moreover, the results of the study of Moser et al. in Paraná showed that 50% of the students in the age range of 18 to 20 and 70% of those 21 to 24 years of age were sexually active (13). Drug abuse and risky sexual behaviors are of the most important dangerous behaviors among students that have exposed the individuals and the society at the risk of dangerous infectious diseases such as AIDS and viral hepatitis (14, 15). These behaviors have reduced years of life due to disability and have been the cause of death of 9.2 million individuals worldwide (16). In our country, it seems that high risk behaviors do not have a high prevalence due to rooted and deeply religious beliefs; however, the evidence shows that in our country, like other countries, the incidence of risky behaviors follows an upward trend (17, 18). Studies have shown that dormitory life, being away from family, unemployment, lack of healthy recreation, and failure to meet emotional needs, and marital status can affect the prevalence of risky behaviors (19).

Given the importance of physical and mental health of students, who constitute a large volume of young population of the country, and considering their professional role and the need for safe staffing to lead the country and their social status as an educated class, their lifestyle not only affects their own personal life, but also influences the behavior and life of other individuals in the society. Because prevention is the best approach to reduce threatening health behaviors at the community level, this study was conducted to investigate the prevalence of risky behaviors among students of Dezful University of Medical Sciences in 2014.

## Materials and Methods

***Study Design and Participants***

This was a descriptive-analytical cross sectional study that included equally 300 male and female students of Dezful University of Medical Sciences.

Required sample size was calculated to be 273 with assumption of 30% risky behaviors prevalence with 5% error. Then, the final sample size was determined as 300 considering 10% dropout. Cluster random sampling was used to select the participants; the sample size was determined from any field of study in accordance with the number of students of that field, and the number of samples was specified in accordance with the percentage of each gender in the fields of study based on the percentage of gender in academic groups. 

***Measuring Tools***

Risky Behaviors Questionnaire was used for data collection. Young's Risk Behaviors Scale (YRBS), which have been borrowed from Risky Behaviors Questionnaire of Control Disease Center of the United States in 1989, was used to evaluate risky behaviors (20).

In this study, IARS (Iranian Adolescents Risk-Taking Scale) was made by investigating valid and proposed tools in risk-taking areas such adolescent’s Risk-Taking (ARQ) Questionnaire and Control System Questionnaire of Youth Risky Behaviors (YRBSS) by considering cultural requirements and social restrictions of Iran society. Using this questionnaire, we evaluated the severity of risky behaviors in the past 3-month period in the areas of violence (carrying bladed weapons and participating in physical disputes), smoking, consumption of alcoholic beverages, drugs and psychoactive drugs, risky sexual relations, nutrition and physical activity, and bad friends. Respondents expressed their agreement and opposition to the statements in a scale of 5 options from completely agree (= 5) to completely disagree (=1). (IARS) Iranian Adolescents Risk-Taking Scale Validity has been investigated by method of internal harmony and help of Cronbach's alpha, and its structure validity was done using exploratory factor analysis and method of analyzing main components. In the questionnaire, there 6 questions pertained to high speed driving (minimum score of 6 and maximum of 30), 5 questions to maim and offense (minimum score of 5 and maximum of 25), 19 questions to tobacco use (minimum score of 19 and maximum of 95), and 8 questions to sexual relationships (minimum score of 8 and maximum of 40). Thus, the scores were classified in 3 groups of low, medium, and high. The reliability of this questionnaire was also obtained to be 0.79 by Cronbach's alpha for the total scale of risky behaviors by performing on 377 individuals by Mehrabi et al. in Iran (21). The reliability of this questionnaire was obtained to be in the range of 23.6% to 90.5% using test-retest method with an interval of 2 weeks and by calculating Kappa coefficient for all items by Berner et al. (22). The validity of the questionnaire was also determined by content validity method. After collecting information, the data were entered into the Stata-12 software. 

***Statistical Methods***

Descriptive indicators including the mean and standard deviation were used to analyze data. Chi-square tests, t test, and ANOVA were used for analytical indicators. Significance level was considered less than 0.05.

***Ethical Considerations***

This study was approved by research council of Dezful University of Medical Sciences (DUR123) in 2014.

## Results

In this study, 150 male students and 150 female students were recruited. The results of the study showed that nursing course had the most frequency (41 individuals, 27.3%) among female students, and emergency medical care course had the most frequency (57 individuals, 38%) among male students. The results of the study revealed that most students were in 20 to 24 years age group. The majority of the students (70%) had 4 to 6 family members, and parental education was diploma in 92% of the participants. With respect to parental occupation, the most frequent father's occupation (122 individuals, 40.7%) was self-employed, and 90% of the mothers were housewives. With respect to risky behaviors, 49.7% of the students reported moderate speed driving; of them, 87.7% had reported little drug consumption and also 60% reported little sexual activities (Table 1). The results of the study revealed a significant difference between the mean scores of risky behaviors, high speed driving, assaults, drug consumption, and sexual behaviors among male and female students (p˂0.05) (Table 2). However, the mean scores in various age groups did not show any statistically significant difference (p˃0.05). In investigating various fields of study, the results revealed a significant difference between the mean scores of risky behaviors, high speed driving, drug consumption, and various fields of study (p˂0.05) (Table 3).

The post hoc test results showed that the mean scores of students' risky behaviors in the fields of medical emergency, laboratory sciences, and anesthesiology majors were more than the mean scores of risky behaviors of students of surgery room, nursing, and medical majors. 

 Moreover, the results showed a significant difference between the mean scores of sexual behaviors among students with different grades, and no statistically significant difference was obtained between students of different grades in other risky behaviors (p˃0.05).

In investigating the relationship between the prevalence of risky behaviors with parental education and household size, the results showed no statistically significant relationship between risky behaviors and household size.

**Table1:** Frequency Distribution of Risky Behaviors among Students

**Risky Behaviors**	**Frequency (Percentage)**		
	**high**	**Moderate**	**low**
High speed driving	66 (22.0)	149 (49.7)	85 (28.3)
Assaults and violations	33 (11.0)	94 (31.3)	173 (57.7)
Drug consumption	11 (3.7)	26 (8.7)	263 (87.7)
Sexual behaviors	45 (15.0)	75 (25.0)	180 (60.0)

**Table2:** Mean Score of Risky Behaviors by Gender of Students

**Risky Behaviors**	**Mean ±SD**		**(t test)**	**p-value**
	**male**	**female**		
High speed driving	22.04±6.3	20.16±6.3	2.57	0.011
Assaults	10.89±4.1	9.82±4	2.25	0.025
Drug consumption	30.59±14.3	25.79±12.8	3.04	0.003
Sexual behaviors	20.05±8.4	12.27±5.9	9.26	0.001

**Table3:** Difference between Mean Score of Risky Behaviors According Field of Study

**Variable**	**Mean Square**	**Sum of Square**	**F test**	**p-value**
High speed in driving	70.43	352.15	1.75	**0.12**
Assaults	36.22	181.12	2.18	**0.056**
Drug consumption	480.70	2403.53	2.85	**0.026**
Sexual behaviors	240.78	1203.92	3.71	**0.003**

However, a significant relationship was found between assaults and violations and parental education (p˂0.05). Thus, it can be concluded that the higher the level of parental education, the less violation and assaults is observed among students. Also, the results showed that father's occupation had an inverse significant relationship with risky behaviors, and drug consumption and sexual behavior of students were influenced by father's occupation (p˂0.05), while mother's occupation was not effective in this regard (p˃0.05).

## Discussion

The present study aimed to investigate the prevalence of risky behaviors among students of Dezful University of Medical Sciences in 2014. The prevalence rate of drug abuse in this study was low. Also, in the study of Rahmati et al., small number of students had reported drug consumption during the past year (23). In a survey conducted on students of Kermanshah, 3.9% of students had a history of drug consumption in the past 3 months (24). In a study conducted in Jahrom, 36.2% of students had reported drug consumption at least once (25). In a national study conducted in the United States, 21.7% of students had consumed marijuana (26). In a study conducted in Eldoret, 69.8% drug consumption was also reported, however, it should be noted that the consumption of alcohol, drugs and cigarettes were in this category, and the maximum amount was related to alcohol (27). Studies have also shown the increases in percentages of students' use of marijuana (28). Considering sexual behaviors, the prevalence of sexual behaviors among females was low, while the prevalence of sexual behaviors was not judicable among males, and 25.3 of males reported high level sexual behavior and 36.7 of the males reported moderate sexual behavior. However, in the study conducted by Rahmati et al. a small number of male and female students reported sexual relations (23). The results of this study differs from studies conducted outside of Iran (29), and a national study conducted in the United States also showed that 41.2% of adolescents have had some type of sex (26). Considering the cultural context of studies and cultural differences between Iran and other countries, and considering some sexual behaviors are considered a norm in some countries (30), it seems that these differences can lead to not reporting sexual relations correctly by Iranians.

The results of the present study revealed that female students reported less risky behaviors than male students, while no difference was observed between risky behaviors among males and females in the study conducted by Rahmati et al. on students in Tehran (23). Because living environment influences the human behavior, the difference among studies can be due to different environmental conditions and to society and cultural differences.

 In a study conducted on students of Zahedan, females had more risky behaviors regarding diet than males (31). Also, in another study in Delhi, females had more inappropriate circumstances in nutritional risky behaviors such as drinking little water, while males reported more risky behaviors regarding consumption of tobacco and alcohol (32). Also in the United States, risky behaviors among males have been seen more than in females (33).

In this study, the most frequent occupation of participants’ fathers were self-employed, employee, and unemployed; moreover, fathers’ occupation had a significant inverse relationship with risky behaviors, drug consumption, and sexual behavior of students. The results showed that risky behaviors were less in students whose fathers were teachers, doctors, or engineers, so the reason could be the high level of fathers' education. Fathers who have higher education are more knowledgeable and train their children to decrease their risky behaviors. However, mothers’ occupation did not have any effects in this regard, and this was consistent with the study conducted on students in Zahedan (31). In the study on violence, low physical activity and robbery were risky behaviors in relation to father's occupation, while in our study; drug consumption and sexual behavior were associated with father's occupation. Also, in a study conducted on students of military and civilian families, living in a military family was a protective factor for risky behaviors; in fact, this study showed a relationship between job and risky behaviors (34). In the studies conducted abroad, classification of occupation has not been conducted in the style of Persian articles, but 21 out of 23 studies conducted during the years 1980 to 2012 showed that nonstandard work programs affect the final growth of children, and this condition was more among vulnerable families and parents who were working full-time (35); and with respect to occupation, studies have also shown that the loss of jobs by the mother or father had negative consequences on educational status of students, their behavior at school and university (36), and this was largely in line with the results of our study.

Conducting further studies on students’ behavior and the fields of its incidence in the cultural context of Iran and comparing it with students of other parts of the country, and its relationship with sociocultural variables are highly suggested.

## Limitation

Questions about risky sexual behaviors were limited due to moral excuses, and it should be noted when generalizing the results of this study to other risky behaviors.

## Conclusion

The prevalence of risky behaviors among students of Dezful University was relatively low, and the prevalence of these behaviors in females was far less than the male students. Risky behaviors were not associated with background variables, exception for father's occupation.

## References

[1] Eccles JS, Midgley C, Wigfield A, Buchanan CM, Reuman D, Flanagan C (1993). Development during adolescence. The impact of stage-environment fit on young adolescents' experiences in schools and in families. Am Psychol.

[2] Amitai M, Apter A (2012). Social aspects of suicidal behavior and prevention in early life: a review. Int J Environ Res Public Health.

[3] Khazaei Z, Khazaei S, Valizadeh R, Mazharmanesh S, Mirmoeini R, Mamdohi S (2016). The Epidemiology of Injuries and Accidents in Children Under one Year of Age, during (2009-2016) in Hamadan Province, Iran. Int J Pediatr.

[4] Umberson D, Crosnoe R, Reczek C (2010). Social Relationships and Health Behavior Across Life Course. Annu Rev Sociol.

[5] Sturm R (2002). The effects of obesity, smoking, and drinking on medical problems and costs. Health Aff (Millwood.

[6] Khazaei S, Mazharmanesh S, Khazaei Z, Goodarzi E, Mirmoini R, Mohammadian-Hafshejani A (2016). [An epidemiological study on the incidence of accidents in the Hamadan province during 2009 to 2014 (Persian)]. Pajouhan Scientific Journal.

[7] Lee CW, Kwak NK (2011). Strategic Enterprise Resource Planning in a Health-Care System Using a Multicriteria Decision-Making Model. J Med Syst.

[8] Hindin MJ, Fatusi AO (2009). Adolescent sexual and reproductive health in developing countries: an overview of trends and interventions. Int Perspect Sex Reprod Health.

[9] Guttmacher S, Kelly PJ, Ruiz-Janecko Y (2010). Community-Based Health Interventions.

[10] Metintaş S, Sariboyaci M, Nuhoğlu S, Metintaş M, Kalyoncu C, Etiz S (1998). Smoking patterns of university students in Eskişehir, Turkey. Public Health.

[11] Tehran Municipipality (2012-2013). Action Report of Director Genaral Ofiice for Health.

[12] Teddle C, Tashakkori A (2009). Foundations of mixed methods research: integrating quantitatives and qualitatives appraches in the social and behavioral sciences.

[13] Moser AM, Reggiani C, Urbanetz A (2007). [Risky sexual behavior among university students in health science courses]. Rev Assoc Med Bras.

[14] Cooper ML (2002). Alcohol use and risky sexual behavior among college students and youth: evaluating the evidence. J Stud Alcohol Suppl.

[15] Rakhshani F, Sepehri Z, Keikha M, Rakhshani T, Ebrahimi M (2011). Paan Use in South-Eastern Iran: The Associated Factors. Iran Red Crescent Med J.

[16] Larki M TM, Latifnejad Roudsari M, Babaee A (2015). The Impact of an Educational Program on Knowledge and Attitude of Female Sex Workers in Preventing High Risk Sexual Behaviours. J Midwife Reproductive health.

[17] Ghaderi M, Ahmadi Z, Darabzadeh F, Nasiri M, Fakouri E (2015). [Correlation between Emotional Intelligence and Risky Sexual Behaviors in Nursing Students of Khozestan Province Universities in 2013 (Persian)]. J Clin Res Paramed Sci.

[18] Hedayati-Moghaddam MR, Eftekharzadeh-Mashhadi I, Fathimoghadam F, Pourafzali SJ (2015). Sexual and Reproductive Behaviors among Undergraduate University Students in Mashhad, a City in Northeast of Iran. J Reprod Infertil.

[19] DuRant RH, Smith JA, Kreiter SR, Krowchuk DP (1999). The relationship between early age of onset of initial substance use and engaging in multiple health risk behaviors among young adolescents. Arch Pediatr Adolesc Med.

[20] Dehghani Z, Zareh A, Dehghani H, Sedghi H, Poormuvahed Z (2010). [Prevalence and factors associated with drug abuse among students of Yazd University of Medical Sciences (persion)]. Journal of Shaheed Sadoughi University of Medical Sciences.

[21] Mehrabi H, Kajbaf M, Mojahed A, Faculty of Education and Psychology, University of Al-Zahra (2011). [Forecast of sensation seeking and risk-taking behaviors based on demographic factors of the students.

[22] Brener ND, Collins JL, Kann L, Warren CW, Williams BI (1995). Reliability of the Youth Risk Behavior Survey Questionnaire. Am J Epidemiol.

[23] Rahmati-Najarkolaei F, Kamalikhah T, Goldoust-Marandy F, Mohammadreza J (2014). A Comparative Study of Health-risk Behaviors of Boys and Girls of Freshmen Year at Tehran University, Iran. Iran J Health Sci.

[24] Jalilian F, Karami Matin B, Ahmadpanah M, Ataee M, Ahmadi Jouybari T, Eslami AA (2015). Socio-demographic characteristics associated with cigarettes smoking, drug abuse and alcohol drinking among male medical university students in Iran. J Res Health Sci.

[25] Heydari ST, Izedi S, Sarikhani Y, Kalani N, Akbary A, Miri A (2015). The Prevalence of Substance use and Associated Risk Factors Among University Students in the City of Jahrom, Southern Iran. Int J High Risk Behav Addict.

[26] Kann L, McManus T, Harris WA, Shanklin SL, Flint KH, Hawkins J (2016). Youth Risk Behavior Surveillance - United States, 2015. MMWR Surveill Summ.

[27] Atwoli L, Mungla PA, Ndung'u MN, Kinoti KC, Ogot EM (2011). Prevalence of substance use among college students in Eldoret, western Kenya. BMC Psychiatry.

[28] Mohler-Kuo M, Lee JE, Wechsler H (2003). Trends in marijuana and other illicit drug use among college students: results from 4 Harvard School of Public Health College Alcohol Study surveys: 1993–2001. J Am Coll Health.

[29] Caico C (2014). Sexually risky behavior in college-aged students. Open Journal of Preventive Medicine.

[30] Timmerman G, Bajema C (2000). The impact of organizational culture on perceptions and experiences of sexual harassment. Journal of Vocational Behavior.

[31] Raghibi M (2012). Examining high risk behaviors among students of Zahedan Universities. Int J High Risk Behav Addict.

[32] Rustagi N, Taneja D, Mishra P, Ingle G (2011). Cardiovascular Risk Behavior among Students of a Medical College in Delhi. Indian J Community Med.

[33] Croisant SA, Haque Laz T, Rahman M, Berenson AB (2013). Gender differences in risk behaviors among high school youth. Glob Adv Health Med.

[34] Tahour-Soltani A (2015). The Comparison of Risky Behaviors and Some of Their Underlying Factors in the College Students from Military and Non-Military Families. Journal Mil Med.

[35] Jafari Mianaei S, Alaei Karahroudi F, Rasouli M (2013). [Study of the impacts of rehabilitation program on mothers with premature hospitalized infants (Persian)]. Education & Ethic In Nursing.

[36] Warr P, Jackson P, Banks M (1988). Unemployment and mental health: Some British studies. Journal of social issues.

